# Upregulated and Hyperactivated Thalamic Connexin 43 Plays Important Roles in Pathomechanisms of Cognitive Impairment and Seizure of Autosomal Dominant Sleep-Related Hypermotor Epilepsy with S284L-Mutant α4 Subunit of Nicotinic ACh Receptor

**DOI:** 10.3390/ph13050099

**Published:** 2020-05-18

**Authors:** Kouji Fukuyama, Masashi Fukuzawa, Motohiro Okada

**Affiliations:** 1 Department of Neuropsychiatry, Division of Neuroscience, Graduate School of Medicine, Mie University, Tsu, Mie 514-8507, Japan; k-fukuyama@clin.medic.mie-u.ac.jp; 2 Department of Biology, Faculty of Agriculture and Life Science, Hirosaki University, Hirosaki 036-8560, Japan; fukuzawa@hirosaki-u.ac.jp

**Keywords:** idiopathic epilepsy, zonisamide, carbamazepine, cognition, connexin, hemichannel

## Abstract

To understand the pathomechanism and pathophysiology of autosomal dominant sleep-related hypermotor epilepsy (ADSHE), we studied functional abnormalities of glutamatergic transmission in thalamocortical pathway from reticular thalamic nucleus (RTN), mediodorsal thalamic nucleus (MDTN) to orbitofrontal cortex (OFC) associated with S286L-mutant α4β2-nicotinic acetylcholine receptor (nAChR), and connexin43 (Cx43) hemichannel of transgenic rats bearing rat S286L-mutant *Chrna4* gene (S286L-TG), corresponding to the human S284L-mutant *CHRNA4* gene using simple Western analysis and multiprobe microdialysis. Cx43 expression in the thalamic plasma membrane fraction of S286L-TG was upregulated compared with that of wild-type. Subchronic administrations of therapeutic-relevant doses of zonisamide (ZNS) and carbamazepine (CBZ) decreased and did not affect Cx43 expression of S286L-TG, respectively. Upregulated Cx43 enhanced glutamatergic transmission during both resting and hyperexcitable stages in S286L-TG. Furthermore, activation of GABAergic transmission RTN–MDTN pathway conversely enhanced, but not inhibited, l-glutamate release in the MDTN via upregulated/activated Cx43. Local administration of therapeutic-relevant concentration of ZNS and CBZ acutely supressed and did not affect glutamatergic transmission in the thalamocortical pathway, respectively. These results suggest that pathomechanisms of ADSHE seizure and its cognitive deficit comorbidity, as well as pathophysiology of CBZ-resistant/ZNS-sensitive ADSHE seizures of patients with S284L-mutation.

## 1. Introduction

Numerous mutations of genes encoding various ion channels, which regulate transmission in the central nervous system, were identified in the various idiopathic epilepsy pedigrees. The functional abnormality of mutant ion channels has been demonstrated by Xenopus oocytes or human embryonic kidney cells, whereas the cellular functional abnormalities cannot sufficiently resemble the situation in vivo epileptic brain [1]. In other words, neither pathomechanisms nor pathophysiologies of any idiopathic epilepsy syndromes have been generated by only functional abnormalities of ion channels [1]. Autosomal dominant sleep-related hypermotor epilepsy (ADSHE), the first identified as a distinct familial idiopathic epilepsy (previously, ADNFLE: autosomal dominant nocturnal frontal lobe epilepsy) in 1994 [2,3], was reported as a channelopathy caused by a mutation in the *CHRNA4* gene, which encodes α4 subunit of nicotinic acetylcholine receptor (nAChR). Until recently, various mutations in several genes such as *CHRNA2, CHRNA4, CHRNB2, CHR, KCNT1*, and *DEPDC5* have been identified in various pedigrees of ADSHE [3,4,5]. ADSHE seizures are symptomatically comparable to those seen in frontal lobe epilepsy and usually occur during the non-rapid eye movement sleep phase [3,4,5,6]. Therefore, any clinical phenotypes have been considered to be uniform to ADSHE syndrome [3]. In spite of uniformity, ADSHE is classified based on the characteristics in two major clinical variations, anticonvulsants sensitivity and cognitive deficit comorbidity [3,4,5,6]. The first-choice anticonvulsant against ADSHE, carbamazepine (CBZ), improves prognosis, and approximately 60% of ADSHE patients remission, including ADSHE patients with S280F and insL mutations of *CHRNA4* [6,7,8], whereas ADSHE patients with S284L-mutation of *CHRNA4* are usually resistant to CBZ, but improved by other anticonvulsants such as zonisamide (ZNS) [4,9,10,11,12]. ADSHE seizures are usually the sole major symptom of the majority of ADSHE patients. Indeed, additional neuropsychiatric features have been reported in just lower than 3% of ADSHE patients [5,13,14,15,16,17]. On the contrary, ADSHE with insL and S284L mutations comorbid with cognitive dysfunction, including schizophrenia-like psychosis, autism, and intellectual disability [10,11,12,15,18,19,20].

Recently, we have demonstrated the pathomechanisms of ADSHE seizures such as nocturnal paroxysmal dystonia, nocturnal paroxysmal arousal and episodic nocturnal wandering, and cognitive impairment, as well as pathophysiology of CBZ-resistant/ZNS-sensitive ADSHE seizures, using a genetic ADSHE model rat, namely S286L transgenic rat (S286L-TG), bearing the missense S286L-mutation in the rat *Chrna4* gene, which corresponds to the S284L-mutation in the human *CHRNA4* [21,22,23]. The functional abnormality of S284L-mutant α4β2-nAChR comprises an enhancement of ACh sensitivity with desensitisation. The combination of these two functional abnormalities leads to loss-of-function of S284L-mutant α4β2-nAChR [24,25], which contributes to the development of several pathomechanisms of ADSHE with S284L-mutation. Basal extracellular l-glutamate level in various brain regions such as mediodorsal (MDTN) and motor (MoTN) thalamic nuclei, secondary motor (M2C) and orbitofrontal (OFC) cortexes, and subthalamic nucleus and substance nigra pars compacta of S286L-TG were larger compared with wild-type rats [21,22,23,26]. Activation of S286L-mutant α4β2-nAChR in the reticular thalamic nucleus (RTN) of S286L-TG produced the relative GABAergic disinhibition in the MoTN, resulting in enhancement of glutamatergic transmission in the thalamocortical motor pathway (MoTN–M2C) [21,23], as well as in the thalamic hyperdirect pathway (MoTN–STN) [22]. The hyperactivation of the thalamic hyperdirect pathway plays important role in the generation of electroencephalogram insensitive nocturnal paroxysmal dystonia, which is a major symptom of ADSHE as paroxysmal movement disorder [22]. Contrary to the thalamic hyperdirect pathway, the M2C itself cannot independently generate epileptic discharge, but can integrate external excitatory inputs from the thalamocortical motor pathway (MoTN–M2C), leading to proceeding epileptic focus [21]. The mechanisms of integration of inputs are modulated by upregulated astroglial connexin 43 (Cx43) and its associated hemichannel, which is induced by loss-of-function of S286L-mutant α4β2-nAChR [23]. These demonstrated functional abnormalities explain the pathomechanisms of three typical ADSHE seizures phenotypes, “nocturnal paroxysmal arousals”, “nocturnal paroxysmal dystonia”, and “episodic nocturnal wandering” [21,22].

In spite of these efforts, the pathomechanisms of cognitive deficit comorbidity of ADSHE with S284L-mutation remain to be clarified. In our previous study, the functional abnormalities of regulatory mechanisms of intrathalamic GABAergic transmission between the motor (MoTN–M2C) and cognitive (MDTN–OFC) glutamatergic pathway were not identical [21]. In particular, activation of α4β2-nAChR in RTN suppresses neuronal activity in both MoTN and MDTN via enhanced GABAergic transmission; however, loss-of-function of S286L-mutant α4β2-nAChR in RTN leads to GABAergic disinhibition in both MoTN and MDTN [21]. The GABAergic disinhibition in the MoTN generates hyperactivation of transmission in the thalamocortical motor pathway, whereas GABAergic disinhibition in the MDTN limits to inactivation, but does not converse to activation of glutamatergic transmission in the thalamocortical cognitive pathway (MDTN–OFC). The incomplete abnormality in MDTN from RTN inhibitory input induced by S286L-mutant α4β2-nAChR possibly contributes to pathomechanisms of cognitive deficit of ADSHE with S284L-mutation [21]. It has been well known that OFC is one of the major epileptic focus regions of ADSHE [3,4,5,6]. Additionally, astroglial Cx43 plays important roles in the implication of cognition and behaviour [27,28,29]. Reduced Cx43 expression was absorbed in the frontal cortex of suicide complete individuals comorbid depression/alcoholism patients [27,28]. Preclinical studies also demonstrated that attenuation of Cx43 function in rodents exhibited anxiolytic/depressive-like behaviours, and exposure to inescapable aversive conditions reduced astroglial Cx43 expression of rats [30,31]. Furthermore, a gold-standard antipsychotic against treatment-refractory schizophrenia, clozapine chronically increased Cx43 expression in the astroglial plasma membrane [29]. On the basis of these clinical and pre-clinical findings, to explore the unresolved issue regarding the functional abnormalities of transmission in the thalamocortical cognitive pathway, we determined the regulation mechanisms of transmission in the RTN–MDTN–OFC pathway associated with connexin hemichannel, as both frontal cortex and thalamus are the most predominant α4β2-nAChR expression regions [21,26].

## 2. Results

### 2.1. Effects of Subchronic Administrations of Therapeutic-Relevant Doses of CBZ and ZNS on Cx43 Expression in the Thalamic Cytosol and Plasma Membrane Fractions of S286L-TG

**Figure 1:**
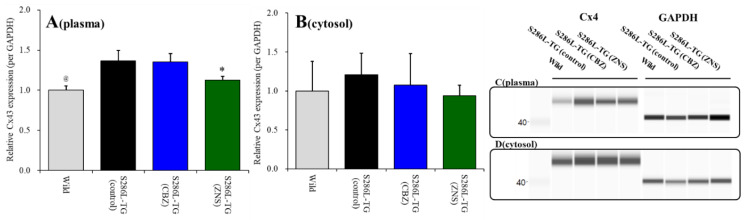
Effects of subcutaneously subchronic administration of therapeutic-relevant doses of carbamazepine (CBZ) and zonisamide (ZNS) (25 mg/kg/day) for seven days on connexin 43 (Cx43) expression in the thalamic plasma membrane (panel **A**) and cytosol (panel **B**) fractions. The pseudo-gel images using simple Western results using anti-glyceraldehyde-3-phosphate dehydrogenase (GAPDH) and anti-Cx43 antibody for blotting of plasma membrane (panel **C**) and cytosol (panel **D**) fractions. In panels 2A and 2B, ordinate: mean ± SD (*n* = 6) of relative protein level of Cx43. @ *p* < 0.05 relative to control by student’s *t*-test, and * *p* < 0.05 relative to control by one-way analysis of variance (ANOVA) with Tukey’s *post hoc* test.

Cx43 expression in the thalamic plasma membrane fraction of S286L-TG was larger than those of wild-type (*p* < 0.05) (Figure 1A). Cx43 expression in the thalamic plasma membrane fraction was decreased by subchronic administration of the therapeutic-relevant dose of ZNS (25 mg/kg/day for seven days), but not by that of CBZ (25 mg/kg/day) [F(2,15) = 11.1 (*p* < 0.01)] (Figure 1A). Contrary to plasma membrane fraction, Cx43 expression in thalamic cytosol fraction between wild-type and S286L-TG were almost equal, and neither subchronic administration of CBZ nor ZNS affected Cx43 expression in thalamic cytosol fraction (Figure 1B).

Therefore, the Cx43 expression in the thalamic plasma membrane of S286L-TG is larger than that of wild-type, similar to M2C [23]. Furthermore, subchronic administration of a therapeutic-relevant dose of ZNS decreased Cx43 expression in the thalamic plasma membrane without affecting that in cytosol, similar to M2C [23].

### 2.2. Effects of Local Administrations of Carbenoxolone (CBX), CBZ, and ZNS into the MDTN on Amino-3-(3-Hydroxy-5-Methyl-Isoxazol-4-yl)propanoic Acid (AMPA)-Evoked l-Glutamate Release in the OFC (Study 1)

**Figure 2:**
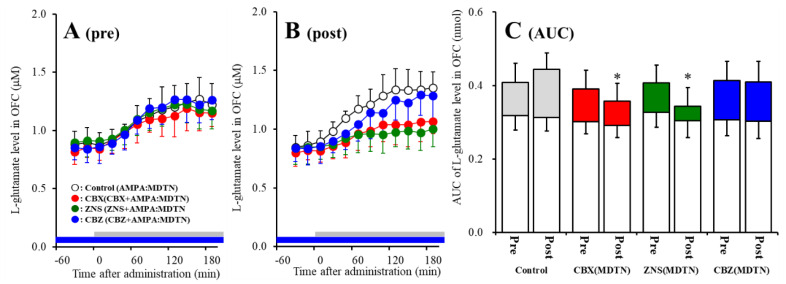
Effects of local administration of 100 μM carbenoxolone (CBX: non-selective hemichannel inhibitor: red circles) and therapeutic-relevant concentration of 100 μM carbamazepine (CBZ: blue circles, estimated concentration in brain tissue is 22 μM) and 500 μM zonisamide (ZNS: green circles, estimated concentration brain tissue is 98 μM) into the mediodorsal thalamic nucleus (MDTN) on pre amino-3-(3-hydroxy-5-methyl-isoxazol-4-yl)propanoic acid (AMPA)-evoked (panel **A**: pre) and post-AMPA-evoked (panel **B**: post) l-glutamate release (before and after hemichannel activation induced by perfusion with Ca^2+^-free with 100 mM K^+^ containing modified Ringer’s solution (FCHK-MRS) into the MDTN for 20 min) in the orbitofrontal cortex (OFC) of wild-type. Ordinates indicate mean extracellular l-glutamate level (μM) (*n* = 6), and abscissas indicate time after AMPA-evoked stimulations (min). Blue bars indicate the perfusion with CBX, CBZ, or ZNS into the MDTN. Gray bars indicate the perfusion with 100 μM AMPA into the MDTN (AMPA-evoked stimulation). (Panel **C**) indicates the area under curve (AUC) value of extracellular l-glutamate level (nmol) before (basal extracellular l-glutamate level) and after AMPA-evoked stimulation (from 20 to 180 min) of panels A and B. Opened columns indicate the AUC values of basal extracellular levels of l-glutamate in panel A and B. * *p* < 0.05; relative to control by multivariate ANOVA (MANOVA) with Tukey’s *post hoc* test.

The detailed experimental design of Study_1 was described in following Section 4.3.1. Perfusion with 100 μM AMPA into the MDTN increased l-glutamate release in the OFC of wild-type (pre-AMPA-evoked release) (Figure 2A). Neither perfusion with 100 μM CBX, 100 μM CBZ, nor 500 μM ZNS into the MDTN affected pre-AMPA-evoked l-glutamate release (Figure 2A,C). Activation induced by perfusion with FCHK-MRS into the MDTN for 20 min (hemichannel activation) did not affect the basal or AMPA-evoked releases (post-AMPA-evoked release) of l-glutamate in the OFC of wild-type (Figure 2A–C). After the hemichannel activation, perfusion with 100 μM CBX and 500 μM ZNS in into the MDTN inhibited post-AMPA-evoked l-glutamate release, but perfusion with 100 μM CBZ did not affect [F_agent_ (3,20) = 4.5 (*p* < 0.05), F_time_ (7.0,140.0) = 90.7 (*p* < 0.01), F_agent*time_ (20.9,140.0) = 6.1 (*p* < 0.01)] (Figure 2B,C).

**Figure 3:**
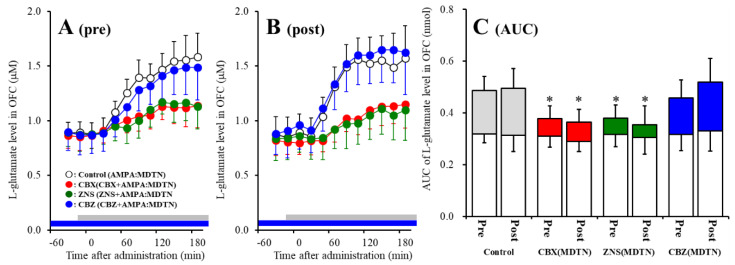
Effects of local administration of 100 μM CBX (red circles) and therapeutic-relevant concentration of CBZ (100 μM: blue circles) and ZNS (500 μM: green circles) into the MDTN on pre-AMPA-evoked (panel **A**) and post-AMPA-evoked (panel **B**) l-glutamate release (before and after hemichannel activation induced by perfusion with FCHK-MRS into the MDTN for 20 min) in the OFC of S286L-TG. Ordinates indicate mean extracellular l-glutamate level (μM) (*n* = 6), and abscissas indicate time after AMPA-evoked stimulations (min). Blue bars indicate the perfusion with CBX, CBZ, or ZNS into the MDTN. Gray bars indicate the perfusion with 100 μM AMPA into the MDTN. (Panel **C**) indicates AUC value of extracellular l-glutamate level (nmol) before (basal extracellular l-glutamate level) and after AMPA-evoked stimulation (from 20 to 180 min) of panels A and B. Opened columns indicate the AUC values of basal extracellular levels of l-glutamate in panel A and B. * *p* < 0.05; relative to control by MANOVA with Tukey’s *post hoc* test.

Similar to wild-type, perfusion with 100 μM AMPA into the MDTN increased l-glutamate release in the OFC of S286L-TG before and after hemichannel activation (Figure 3). Activated hemichannel induced by perfusion with FCHK-MRS into the MDTN for 20 min (hemichannel activation) did not affect the basal or AMPA-evoked releases (post-AMPA-evoked release) of l-glutamate in the OFC of S286L-TG (Figure 3A–C).

Contrary to wild-type, both perfusion with 100 μM CBX and 500 μM ZNS into the MDTN inhibited pre-AMPA-evoked [F_agent_ (3,20) = 4.8 (*p* < 0.05), F_time_ (4.7,94.7) = 217.4 (*p* < 0.01), F_agent*time_ (14.2, 94.7) = 13.8 (*p* < 0.01)] and post-AMPA-evoked l-glutamate releases [F_agent_ (3,20) = 7.1 (*p* < 0.01), F_time_ (6.6,132.4) = 147.3 (*p* < 0.01), F_agent*time_ (19.9,132.4) = 10.6 (*p* < 0.01)], but perfusion with 100 μM CBZ into the MDTN did not affect those of S286L-TG (Figure 3A–C).

The results in study 1 indicate that extracellular astroglial plasma extramembrane condition complex among membrane depolarization, increased extracellular potassium level, and decreased extracellular calcium level activate hemichannel in the MDTN. Additionally, hemichannel associated thalamocortical glutamatergic transmission of S286L-TG is more dominant compared with that of wild-type during both the resting and hyperactivated state.

### 2.3. Effects of Local Administrations of (E)-N-Methyl-4-(3-Pyridinyl)-3-Buten-1-Amine Oxalate (RJR2403) into the RTN on AMPA-Evoked l-Glutamate Release in the OFC (Study 2)

**Figure 4:**
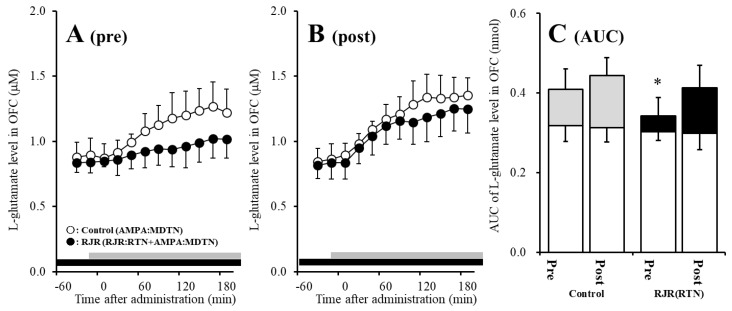
Effects of local administration of 100 μM (E)-*N*-Methyl-4-(3-pyridinyl)-3-buten-1-amine oxalate (RJR2403) (RJR: selective α4β2-nAChR agonist: closed circles) into the RTN on pre-AMPA-evoked (panel **A**) and post-AMPA-evoked (panel **B**) l-glutamate release (before and after hemichannel activation induced by perfusion with FCHK-MRS into the MDTN for 20 min) in the OFC of wild-type. Ordinates indicate mean extracellular l-glutamate level (μM) (*n* = 6), and abscissas indicate time after AMPA-evoked stimulations (min). Closed bars indicate the perfusion with RJR2403 into the RTN. Gray bars indicate the perfusion with 100 μM AMPA into the MDTN. (Panel **C**) indicates the AUC value of the extracellular l-glutamate level (nmol) before (basal extracellular l-glutamate level) and after AMPA-evoked stimulation (from 20 to 180 min) of panels (**A**,**B**). Opened columns indicate the AUC values of basal extracellular levels of l-glutamate in panel (**A**,**B**). * *p* < 0.05; relative to control by MANOVA with Tukey’s post hoc test. The control data were the same data in study 1 (Figure 2).

The detailed experimental design of Study_2 was described in following Section 4.3.2. Before hemichannel activation in the MDTN, perfusion with 100 μM (E)-*N*-Methyl-4-(3-pyridinyl)-3-buten-1-amine oxalate (RJR2403: selective α4β2-nAChR agonist) into the RTN inhibited pre-AMPA-evoked l-glutamate release in the OFC of wild-type [F_agent_ (1,10) = 5.1 (*p* < 0.05), F_time_ (5.0,49.7) = 37.2 (*p* < 0.01), F_agent*time_ (5.0,49.7) = 6.1 (*p* < 0.01)] (Figure 4A,C). After the hemichannel activation, perfusion with 100 μM RJR2403 into the RTN did not affect post-AMPA-evoked l-glutamate release in the OFC of wild-type (Figure 4B,C).

**Figure 5:**
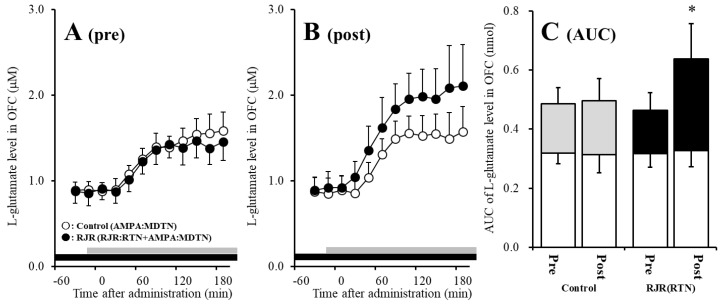
Effects of local administration of 100 μM RJR2403 (RJR: selective α4β2-nAChR agonist: closed circles) into the RTN on pre-AMPA-evoked (panel **A**) and post-AMPA-evoked (panel **B**) l-glutamate release (before and after hemichannel activation induced by perfusion with FCHK-MRS into the MDTN for 20 min) in the OFC of S286L-TG. Ordinates indicate mean extracellular l-glutamate level (μM) (N = 6), and abscissas indicate time after AMPA-evoked stimulations (min). Closed bars indicate the perfusion with RJR2403 into the RTN. Gray bars indicate the perfusion with 100 μM AMPA into the MDTN. (Panel **C**) indicates the AUC value of the extracellular l-glutamate level (nmol) before (basal extracellular l-glutamate level) and after AMPA-evoked stimulation (from 20 to 180 min) of panels (**A**,**B**). Opened columns indicate the AUC values of basal extracellular levels of l-glutamate in panel (**A**,**B**). * *p* < 0.05; relative to control by MANOVA with Tukey’s *post hoc* test. The control data were the same data in Figure 3.

Contrary to wild-type, before activation of hemichannel in the MDTN, perfusion with 100 μM RJR2403 (selective α4β2-nAChR agonist) into the RTN did not affect pre-AMPA-evoked l-glutamate release in the OFC of S286L-TG (Figure 5A,C). After the hemichannel activation, perfusion with 100 μM RJR2403 into the RTN enhanced post-AMPA-evoked l-glutamate release in the OFC of S286L-TG [F_agent_ (1,10) = 5.5 (*p* < 0.05), F_time_ (2.7,26.5) = 136.2 (*p* < 0.01), F_agent*time_ (2.7,26.5) = 6.8 (*p* < 0.01)] (Figure 5B,C).

Study 2 indicates the interesting possibilities that activated hemichannel activity in the MDTN prevents the inhibitory GABAergic input from the RTN induced by α4β2-nAChR activation. Furthermore, activated inhibitory GABAergic input into the MDTN of S286L-TG conversely enhances thalamocortical glutamatergic transmission.

### 2.4. Effects of Local Administrations of CBX and Muscimol (MUS) into the MDTN on RJR-Evoked l-Glutamate Release in the MDTN (Study 3)

**Figure 6:**
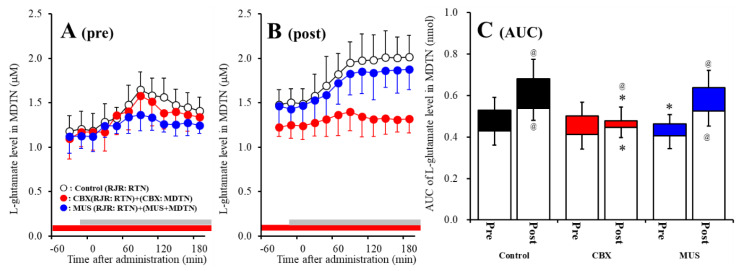
Effects of local administration of 100 μM CBX (non-selective hemichannel inhibitor) and 1 μM muscimol (MUS: GABA_A_ receptor agonist) into the MDTN on pre-RJR- (panel **A**) and post-RJR-evoked (panel **B**) l-glutamate release (before and after hemichannel activation induced by perfusion with FCHK-MRS into the MDTN for 20 min) in the MDTN of wild-type. Ordinates indicate mean extracellular l-glutamate level (μM) (N = 6), and abscissas indicate time after RJR-evoked stimulations (min). Gray bars indicate the perfusion with 100 μM RJR2403 into the RTN. Red bars indicate the perfusion with 100 μM CBX or 1 μM MUS into the MDTN. (Panel **C**) indicates the AUC value of the extracellular l-glutamate level (nmol) before (basal extracellular l-glutamate level) and after RJR-evoked stimulation (from 20 to 180 min) of panels (**A**,**B**). Opened columns indicate the AUC values of basal extracellular levels of l-glutamate in panel (**A**,**B**). * *p* < 0.05 relative to control, @ *p* < 0.05 relative to before hemichannel activation (pre) by MANOVA with Tukey’s *post hoc* test.

The detailed experimental design of Study_3 was described in following Section 4.3.3. Both before and after activation of hemichannel in the MDTN, perfusion with 100 μM RJR2403 (selective α4β2-nAChR agonist) into the RTN increased l-glutamate release in the MDTN of wild-type (Figure 6A–C). Study 3 detected the significant interaction between perfusion with 100 μM CBX (non-selective hemichannel inhibitor) into the MDTN and activation of hemichannel in the MDTN (perfusion with FCHK-MRS into the MDTN for 20 min) on RJR-evoked l-glutamate release in the MDTN of wild-type [F_agent_ (1,20) = 14.1 (*p* < 0.01), F_activation_ (1,20) = 5.2 (*p* < 0.05), F_agent*activation_ (1,20) = 8.1 (*p* < 0.01), F_time_ (7.0,140.0) = 62.5 (*p* < 0.01), F_activation*time_ (7.0,140.0) = 4.8 (*p* < 0.01), F_agent*time_ (7.0,140.0) = 10.5 (*p* < 0.01), F_activation*agent*time_ (7.0,140.0) = 6.9 (*p* < 0.01)] (Figure 6A–C). Activation of hemichannel in the MDTN enhanced RJR-evoked l-glutamate release (Figure 6A–C). Before hemichannel activation, perfusion with 100 μM CBX into the MDTN did not affect basal or pre-RJR-evoked releases of l-glutamate release in the MDTN (Figure 6A,C); however, after the hemichannel activation, perfusion with 100 μM CBX into the MDTN inhibited post-RJR-evoked l-glutamate release in the MDTN (Figure 6B,C).

Study 3 detected the significant interaction between perfusion with 1 μM MUS (GABA_A_ receptor agonist) into the MDTN and activation of hemichannel in the MDTN of wild-type on RJR-evoked l-glutamate release in the MDTN [F_agent_ (1,20) = 3.0 (*p* > 0.05), F_activation_ (1,20) = 29.4 (*p* < 0.01), F_agent*activation_ (1,20) = 0.1 (*p* > 0.05), F_time_ (5.8,115.9) = 76.3 (*p* < 0.01), F_activation*time_ (5.8,115.9) = 14.9 (*p* < 0.01), F_agent*time_ (5.8,115.9) = 4.2 (*p* < 0.01), F_activation*agent*time_ (5.8,115.9) = 1.2 (*p* > 0.05)] (Figure 6A–C). Before hemichannel activation, perfusion with 1 μM MUS into the MDTN did not affect basal l-glutamate release in the MDTN, but inhibited pre-RJR-evoked l-glutamate release in the MDTN (Figure 6C); however, after the hemichannel activation, the inhibitory effect of perfusion with 1 μM MUS into the MDTN on post-RJR-evoked l-glutamate release in the MDTN was abolished (Figure 6A–C).

**Figure 7:**
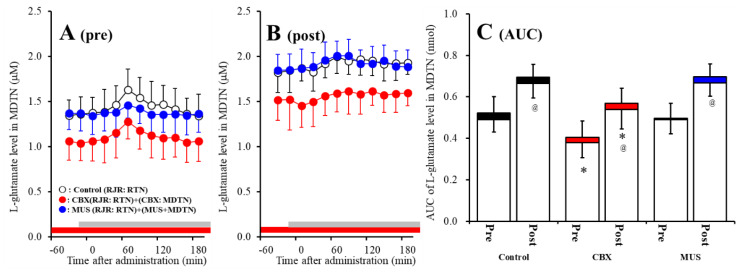
Effects of local administration of 100 μM CBX (non-selective hemichannel inhibitor) and 1 μM MUS (GABA_A_ receptor agonist) into the MDTN on pre-RJR- (panel **A**) and post-RJR-evoked (panel **B**) l-glutamate release (before and after hemichannel activation induced by perfusion with FCHK-MRS into the MDTN for 20 min) in the MDTN of S286L-TG. Ordinates indicate mean the extracellular l-glutamate level (μM) (N = 6), and abscissas indicate time after RJR-evoked stimulations (min). Gray bars indicate the perfusion with 100 μM RJR2403 into the RTN. Red bars indicate the perfusion with 100 μM CBX or 1 μM MUS into the MDTN. (Panel **C**) indicates the AUC value of the extracellular l-glutamate level (nmol) before (basal extracellular l-glutamate level) and after RJR-evoked stimulation (from 20 to 180 min) of panels (**A**,**B**). Opened columns indicate the AUC values of basal extracellular levels of l-glutamate in panel A and B. * *p* < 0.05 relative to control, @ *p* < 0.05 relative to before hemichannel activation (pre) by MANOVA with Tukey’s post hoc test.

Study 3 detected the significant interaction between perfusion with 100 μM CBX into the MDTN and activation of hemichannel in the MDTN on RJR-evoked l-glutamate release in the MDTN of S286L-TG [F_agent_ (1,20) = 16.9 (*p* < 0.01), F_activation_ (1,20) = 32.2 (*p* < 0.01), F_agent*activation_ (1,20) = 0.1 (*p* > 0.05), F_time_ (9,180) = 15.6 (*p* < 0.01), F_activation*time_ (9,180) = 5.1 (*p* < 0.01), F_agent*time_ (9,180) = 0.6 (*p* > 0.05), F_activation* agent*time_ (9,180) = 0.3 (*p* > 0.05)] (Figure 7A–C). Before hemichannel activation, perfusion with 100 μM CBX into the MDTN decreased basal l-glutamate release in the MDTN without affecting pre-RJR-evoked l-glutamate release in the MDTN (Figure 7A,C). After the hemichannel activation, perfusion with 100 μM CBX into the MDTN also decreased basal l-glutamate release in the MDTN without affecting post-RJR-evoked l-glutamate release in the MDTN (Figure 7B,C).

Study 3 detected the significant interaction between perfusion with 1 μM MUS into the MDTN and activation of hemichannel in the MDTN of wild-type on RJR-evoked l-glutamate release in the MDTN [F_agent_ (1,20) = 0.1 (*p* > 0.05), F_activation_ (1,20) = 45.1 (*p* < 0.01), F_agent*activation_ (1,20) = 0.2 (*p* > 0.05), F_time_ (9,180) = 15.3 (*p* < 0.01), F_activation*time_ (9,180) = 1.6 (*p* < 0.01), F_agent*time_ (9,180) = 1.8 (*p* > 0.05), F_activation*agent*time_ (9,180) = 2.1 (*p* < 0.05)] (Figure 7A–C). Before hemichannel activation, perfusion with 1 μM MUS into the MDTN did not affect basal or pre-RJR-evoked releases of l-glutamate in the MDTN (Figure 7A,C). After the hemichannel activation, perfusion with 1 μM MUS into the MDTN did not affect basal or post-RJR-evoked releases of l-glutamate in the MDTN (Figure 7B,C).

Surprisingly, demonstrated in Study 2 (Figure 4 and Figure 5), enhanced thalamocortical glutamatergic transmission induced by GABAergic input from the RTN is probably not associated with the GABA_A_ receptor function, but is possibly affected by neuronal excitability input.

### 2.5. Effects of Local Administration of CBX into the MDTN on RJR-Evoked GABA Release in the MDTN (Study 3)

**Figure 8:**
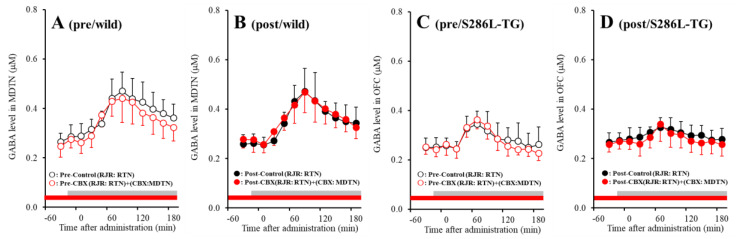
Effects of local administration of 100 μM CBX (non-selective hemichannel inhibitor) into the MDTN on pre-RJR- (panels **A**,**C**) and post-RJR-evoked (panels **B**,**D**) GABA release (before and after hemichannel activation induced by perfusion with FCHK-MRS into the MDTN for 20 min) in the MDTN of wild-type (panels **A**,**B**) and S286L-TG (panels **C**,**D**). Ordinates indicate mean extracellular GABA level (μM) (N = 6), and abscissas indicate time after RJR-evoked stimulations (min). Gray bars indicate the perfusion with 100 μM RJR2403 into the RTN. Red bars indicate the perfusion with 100 μM CBX into the MDTN.

Both before and after activation of hemichannel in the MDTN, perfusion with 100 μM RJR2403 (selective α4β2-nAChR agonist) into the RTN increased GABA release in the MDTN of wild-type and S286L-TG (Figure 8). Before hemichannel activation in the MDTN, perfusion with 100 μM CBX in the MDTN did not affect pre-RJR-evoked GABA release in the MDTN (Figure 8A,C), but the pre-RJR-evoked GABA release in the MDTN of wild-type was larger compared with that of S286L-TG [F_agent_ (1,20) = 0.4 (*p* > 0.05), F_genotype_ (1,20) = 17.9 (*p* < 0.01), F_agent*genotype_ (1,20) = 0.1 (*p* > 0.05), F_time_ (6.1,121.8) = 99.6 (*p* < 0.01), F_genotype*time_ (6.1,121.8) = 24.7 (*p* < 0.01), F_agent*time_ (6.1,121.8) = 4.0 (*p* < 0.01), F_genotype* agent*time_ (6.1,121.8) = 1.4 (*p* > 0.05)] (Figure 8A,C). Similarly, after activation, perfusion with 100 μM CBX in the MDTN also did not affect post-RJR-evoked GABA release in the MDTN (Figure 8B,D), but the post-RJR-evoked GABA release in the MDTN of wild-type was larger compared with that of S286L-TG MDTN [F_agent_ (1,20) = 0.1 (*p* > 0.05), F_genotype_ (1,20) = 14.8 (*p* < 0.01), F_agent*genotype_ (1,20) = 0.3 (*p* > 0.05), F_time_ (4.3,86.3) = 81.1 (*p* < 0.01), F_genotype*time_ (4.3,86.3) = 30.4 (*p* < 0.01), F_agent*time_ (4.3,86.3) = 0.6 (*p* > 0.05), F_genotype*agent*time_ (4.3,86.3) = 2.1 (*p* > 0.05)] (Figure 8B,D).

The stimulatory effects of S286L-mutant α4β2-nAChR on GABAergic neurones in the RTN are impaired compared with that of wild-type. The GABAergic transmission from the RTN to MDTN is insensitive to hemichannel activity in the MDTN.

## 3. Discussion

### 3.1. Pathomechanism of ADSHE Seizures Associated with Cx43

The present study demonstrated the transmission abnormality of S286L-TG in thalamocortical cognitive pathway (RTN-MDTN-OFC) associated with hemichannel and S286L-mutant α4β2-nAChR. Cx43 expression in thalamic plasma membrane fraction of S286L-TG was larger than that of wild-type, resembling M2C [23]. Cx43 is the most widely expressed and predominant expression isoform in the central nervous system and astrocytes among 21 connexin isoforms [32,33]. Although the detailed mechanisms remain to be clarified, it has been demonstrated that the signaling of α4β2-nAChR suppresses the Cx43 expression in vitro and in vivo [23,34]. Connexin constitutes hemichannel/gap-junction, which regulates physiological functions including neuronal excitability, synaptic plasticity, tripartite synaptic transmission, and homeostasis balance in the central nervous system [35,36]. Recently, Cx43 is expected as a novel target for antiepileptic medication, because hemichannel inhibitors supressed the onset of epileptic seizures [36,37,38]. Indeed, similar to S286L-TG, glial Cx43 is increased in the focus region of patients with epilepsy [39]. Astroglial hemichannel exhibits a low opening probability during the resting stage [40,41], but is activated by depolarization of membrane potential and extracellular and intracellular cation levels, increased extracellular K^+^, intracellular Ca^2+^, and decreased extracellular Ca^2+^ levels [40]. Furthermore, once activated, the activation/opening state of hemichannel persists [23]. In the present study, FCHK-MRS (Ca^2+^-free with 100 mM K^+^) stimulation activated hemichannel activity, which was maintained for several hours (Figure 6 and Figure 7). Therefore, toxic hyperexcitable events, such as epileptic seizure, generate elevation of extracellular K^+^ and depletion of extracellular Ca^2+^ [42,43]. The depletion of extracellular Ca^2+^ level suppresses propagation of epileptic hyperexcitability in attenuation of neurotransmission [42]; however, at the same time, combination of astroglial depolarisation and extracellular ionic abnormality induced by epileptic hyperexcitability activates astroglial hemichannel activity [42]. Therefore, the interplay between astroglial depolarization and hemichannel activation produces a further astroglial regenerative activation circular sequence: depolarization activates accelerated increasing cations movements via activated hemichannel, resulting in further depolarizing astroglial membrane potential. Indeed, elevations of extracellular K^+^ and intracellular Ca^2+^ with reduction of extracellular Ca^2+^ generate the releases of excitatory gliotransmitters (i.e., glutamate and ATP), via activated hemichannel [40,41].

**Figure 9:**
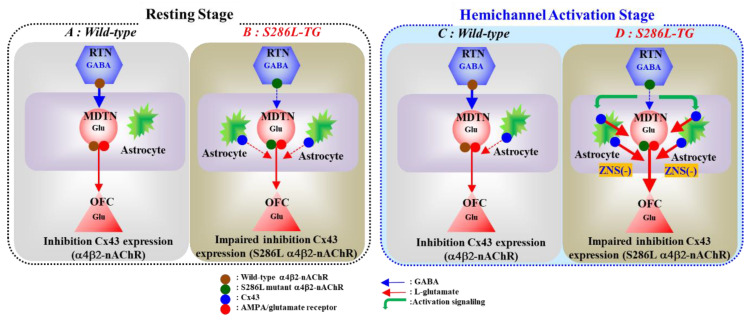
Proposed hypothesis of pathomechanisms and pathophysiology of S286L-TG. Proposed hypothesis for functional abnormalities of glutamatergic transmission in the thalamocortical pathways in wild-type (panel **A**) and S286L-TG (panel **B**) during the resting stage, as well as in wild-type (panel **C**) and S286L-TG (panel **D**) during the hemichannel activation stage. RTN mainly projects GABAergic terminals to MDTN. Activation of α4β2-nAChR in the RTN enhances GABAergic transmission in the RTN-MDTN pathways of wild-type (panel **A**). MDTN project glutamatergic terminals to the OFC. In the MDTN, both α4β2-nAChR and AMPA/glutamate receptor activate glutamatergic transmission to the OFC (panel **A**). Wild-type α4β2-nAChR suppresses Cx43 expression in astroglial plasma membrane in the thalamus (panel **A**). Contrary to wild-type, in S286L-TG, loss-of-function S286L-mutant α4β2-nAChR attenuates the stimulatory and inhibitory effects on respective GABAergic transmission (RTN-MDTN) and Cx43 expression, resulting in upregulated Cx43 in the thalamus (panel **B**). After the activation of hemichannel in the MDTN induced by calcium-free with high potassium stimulation, α4β2-nAChR induced GABA release (RTN-MDTN) is not affected in both wild-type and S286L-TG (panels **C**,**D**). Enhanced astroglial glutamatergic transmission associated with activated hemichannel in the MDTN leads to apparent impairment of GABAergic inhibition in wild-type, similar to S286L-TG during the resting stage (panel **C**). In S286L-TG, propagation of neuronal excitabilities from the RTN to MDTN enhances thalamocortical glutamatergic transmission GABA_A_ receptor independently via increased extracellular potassium and reduced extracellular calcium levels as hemichannel activation signalling (panel **D**). A therapeutic-relevant concentration of ZNS chronically supresses Cx43 expression in the plasma membrane and acutely inhibits hemichannel activity (panel **D**).

Our previous studies demonstrated that impairment of the excitatory effects of S286L-mutant α4β2-nAChR on GABAergic neurons in RTN played important roles in the hyperactivation of thalamocortical and thalamic hyperdirect glutamatergic transmissions [21,22]. Exactly, the inhibitory effect of application of α4β2-nAChR agonist (RJR2403) into the RTN on glutamatergic transmission in the thalamocortical cognitive pathway was abolished in S286L-TG (Figure 5 and Figure 9). The abolishment of GABAergic inhibition on thalamocortical cognitive pathway can be pharmacologically explained by the loss-of-function of S286L-mutant α4β2-nAChR function; however, the detailed mechanisms of relative GABAergic disinhibition induced hyperactivation of thalamocortical motor pathway [21] remain to be clarified. In the present study, the hypothesis the complex between loss-of-function α4β2-nAChR and upregulated Cx43 can successfully clarify the mechanism of enhancement of the thalamocortical motor pathway induced by relative GABAergic disinhibition of S286L-TG.

In wild-type rat, before hemichannel activation, l-glutamate release through hemichannel to basal l-glutamate release in the MDTN during the resting stage was limited (Figure 6 and Figure 9). Activation of hemichannel in the MDTN did not affect GABA release in the MTDN induced by local administration of α4β2-nAChR agonist into the RTN (Figure 8 and Figure 9). Contrary to GABA, activation of hemichannel in the MDTN enhanced l-glutamate release in the MTDN induced by local administration of α4β2-nAChR agonist into the RTN; however, most of the increased l-glutamate release after the hemichannel activation was probably output via CBX sensitive hemichannel (Figure 6 and Figure 9). Furthermore, activation of GABA_A_ receptor in the MDTN did not affect RJR-evoked l-glutamate release under the hemichannel activation. Under the activation of hemichannel, GABA_A_ receptor independent elevation of extracellular l-glutamate level synchronised with increased GABA release in the MDTN induced by activation of GABAergic neuronal activity in the RTN is produced possibly by propagation of depolarisation from the RTN to MDTN. In other words, increased extracellular K^+^ and decreased extracellular Ca^2+^ levels around axon terminals induced by propagation of neuronal excitability [42,43] enhance hemichannel activity and gliotransmitter release via activated hemichannel (Figure 9).

In S286L-TG, the hemichannel in the MDTN of S286L-TG is already activated during the resting stage, because basal l-glutamate release in the MDTN was CBX-sensitive (Figure 7 and Figure 9). Under the activation of MDTN hemichannel activity after the FCHK-MRS stimulation, surprisingly, activation of GABAergic neurones in the RTN (application of RJR2403 into the RTN) enhanced l-glutamate release in the MDTN. Although the excitatory effect of S286L-mutant α4β2-nAChR is attenuated, GABA release in the MDTN could be weakly increased by local administration of α4β2-nAChR agonist (RJR2403) in the RTN. Taken together with the results of wild-type and S286L-TG, therefore, under the upregulated/activated astroglial hemichannel in MDTN of S286L-TG, the excitability propagations, including both excitatory and inhibitory inputs, to the MDTN generate the enhancement of gliotransmitter releases through upregulated/activated astroglial hemichannel. On the basis of these pharmacological experimental results, considering the pathomechanisms of ADSHE seizure, propagation of sleep spindle, a benign physiological discharge during sleep, plays important roles in the generation of ADSHE seizures, probably rather than propagation of S286L-mutant α4β2-nAChR-induced GABAergic activity propagation. Activation of α4β2-nAChR in the RTN of S286L-TG enhanced and did not affect glutamatergic transmission in thalamocortical motor (MoTB-M2C) and cognitive (MDTN-OFC) pathways, respectively [21,23]. After the activation of hemichannel in the MDTN by FCHK-MRS stimulation, activation of α4β2-nAChR in the RTN of S286L-TG enhanced glutamatergic transmission in the thalamocortical cognitive pathway (Figure 3 and Figure 9). These results indicate that persistent or repetitive propagation of discharges to the MDTN possibly generates the hyperexcitability in the thalamocortical cognitive pathway associated with epileptic events. Indeed, several clinical studies reported that the OFC is one of the ADSHE focus regions [5,6,8].

The present study also pharmacologically provides the candidate pathophysiological hypothesis CBZ-resistant/ZNS-sensitive ADSHE seizure of ADSHE with S284L-mutation. The subchronic administration of therapeutic-relevant dose of ZNS decreased upregulated Cx43 in the thalamus of S286L-TG, whereas CBZ had no effect (Figure 1). Additionally, local administration of therapeutic-relevant concentration of ZNS into the MDTN inhibited glutamatergic transmission in the thalamocortical pathway, regardless of activation of hemichannel activity in the MDTN; however, therapeutic-relevant concentration of CBZ had no effect. Therefore, the antiepileptic action of ZNS on ADSHE seizure of patient with S284L-mutation was yielded by the complex between acute suppression of thalamocortical hyperexcitability and chronic compensation of upregulated Cx43 expression (Figure 9).

### 3.2. Pathomechanism of Cognitive Dificit Comorbidity of ADSHE with S284L-Mutation

ADSHE seizure foci have been identified in various frontal lobes and other cortical regions, including motor cortex, OFC, and insula [5]. The glutamatergic neurones in the MDTN project glutamatergic terminals to both OFC and insula [44,45]. Recent clinical studies reported that MDTN dysfunction makes a particularly relevant contribution to cognitive deficits in psychosis [46], intellectual disability [47], autism [46], and epileptic psychosis [48]. MDTN has been considered to regulate the integration of signalling inputs associated with learning, memory, and emotion from various cortical and subcortical regions [49,50,51]. Hyperactivation of thalamocortical glutamatergic transmission via GABAergic disinhibition in the intrathalamic pathway (RTN-NDTN) induced by N-Methyl-D-aspartate (NMDA)/glutamate receptor antagonist plays important roles in the pathophysiology of cognitive deficit in schizophrenia [52]. Aripiprazole, clozapine, guanfacine, lurasidone, amantadine, and memantine improved cognitive dysfunction in various psychotic disorders by compensation of thalamocortical hyperactivation via various distinct mechanisms [44,45,52,53,54,55]. The candidate pharmacological mechanisms of cognitive deficits associated with NMDA hypothesis support the pathomechanisms of cognitive impairment comorbidity in ADSHE patients with S284L-mutation, because, in both pharmacological NMDA/glutamate receptor impairment models and S286L-TG, the thalamocortical glutamatergic hyperfunction induced by GABAergic disinhibition in RTN-NDTN via attenuated excitatory function of NMDA/glutamate receptor and α4β2-nAChR, respectively. Indeed, MDTN-OFC glutamatergic transmission is considered to play important roles in maintaining flexible stimulus-reward associations [45]. On the basis of these previous findings regarding cognitive mechanisms and pathomechanisms of ADSHE seizures, impaired α4β2-nAChR regulation on thalamocortical transmission might contribute to cognitive deficits of ADSHE with S284L-mutation during interictal states.

Astroglial Cx43 has been considered to be implicated in functions of both cognition and behaviour. Reduced Cx43 expression was absorbed in the frontal cortex of suicide complete individual comorbid depression/alcoholism patients [27,28]. Preclinical studies also demonstrated that attenuation of Cx43 function in rodents exhibits anxiolytic/depressive-like behaviours, and exposure to inescapable aversive conditions reduced astroglial Cx43 expression of rats [30,31]. Furthermore, a gold-standard antipsychotic against treatment-refractory schizophrenia, clozapine chronically increased Cx43 expression in the astroglial plasma membrane [29]. Therefore, these previous findings indicate that impaired Cx43 function contributes to cognitive deficits; however, contrary to these findings, Cx43 is upregulated in S286L-TG. This discrepancy suggests the possibility that Cx43 contributes to cognitive function as an essential signalling molecule, but the appropriate Cx43 expression volume and activity for regulation of adequate cognitive processes might exist. Indeed, the Cx43 upregulation pattern of S286L-TG is selective in the frontal cortex [23] and thalamus, which are α4β2-nAChR dominant expression regions [21,26]. Furthermore, the hemichannel constituted by upregulated Cx43 is already activated during the resting stage in S286L-TG. Therefore, the persistent hyperactivation of thalamocortical glutamatergic transmission induced by activated hemichannel in both thalamus and frontal cortex probably degrades the perceptual integration of signalling inputs associated with learning, memory, and emotion from various cortical and subcortical regions. To clarify our hypothesis, we shall report in further studies.

## 4. Materials and Methods

### 4.1. Chemical Agents

The selective α4β2-nAChR agonist, (E)-*N*-Methyl-4-(3-pyridinyl)-3-buten-1-amine oxalate (RJR2403), was obtained from Cosmo Bio (Tokyo, Japan) [56]. Amino-3-(3-hydroxy-5-methyl- isoxazol-4-yl)propanoic acid (AMPA) [56] was obtained from Wako Chemicals (Osaka, Japan). The non-selective hemichannel inhibitor, carbenoxolone (CBX) [29,57], was obtained from Funakoshi (Tokyo, Japan). Carbamazepine (CBZ) was obtained from Tokyo Chemical Industry (Tokyo, Japan). Zonisamide sodium salt (ZNS) was provided by Dainippon-Sumitomo Pharma (Osaka, Japan).

All agents were prepared on the day of the experiment. CBZ was initially dissolved at 50 mM in dimethyl sulfoxide. The final concentration of dimethyl sulfoxide was lower than 0.2% (vol/vol). RJR2403, AMPA, CBX and ZNS were dissolved in modified Ringer’s solution (MRS) or Ca^2+^-free with 100 mM K^+^ containing MRS (FCHK-MRS) directly for microdialysis study. Therapeutic-relevant plasma concentrations of ZNS and CBZ against several convulsion models ranged from 47 to 330 μM and 17 to 42 μM, respectively [58,59]. Accordingly, to explore the effects of therapeutic-relevant concentration of ZNS and CBZ on thalamocortical transmission, 500 μM ZNS (estimated concentration in extracellular fluid 98 μM) and 100 μM CBZ (estimated concentration in extracellular fluid 22 μM) were perfused in dialysate [58,59,60]. The detailed compositions of MRS and FCHK-MRS are described in the following section.

Previous studies have reported that the chronic administration of therapeutic-relevant dose of ZNS and CBZ ranged from 25 to 50 mg/kg/day and 10 to 25 mg/kg/day, respectively [58,61,62,63]. According to previous reports, in the present study, to study the subchronic administrations of therapeutic-relevant doses of ZNS and CBZ on thalamic Cx43 expression, each rat was subcutaneously administered ZNS (25 mg/kg/day for seven days) and CBZ (25 mg/kg/day for seven days) using a subcutaneously osmotic pump (2ML_1, Alzet, Cupertino, CA, USA).

### 4.2. Preparation of the Microdialysis System

Animal care, the experimental procedures, and protocols for animal experiments were approved by the Animal Research Ethics Committee of the Mie University School of Medicine (No. 24–37-R3). All studies involving animals have been reported in accordance with the ARRIVE guidelines for reporting experiments involving animals [64]. A total of 120 rats were used in the experiments described. During subchronic administration of a therapeutic-relevant dose of CBZ and ZNS using osmotic pump, male S286L-TG [21,22,23] and wild-type littermates were anesthetized with 1.8% isoflurane and then placed in a stereotactic frame.

A concentric direct-insertion type dialysis probe (0.22 mm diameter, 3 mm exposed membrane: Eicom, Kyoto, Japan) was implanted in the orbitofrontal cortex (OFC: A = +3.2 mm, L = +2.4 mm, V = −6.5 mm, relative to bregma). Another concentric direct-insertion type probe with a shorter exposed membrane (0.22 mm diameter, 2 mm exposed membrane: Eicom) was then implanted in the mediodorsal thalamic nucleus (MDTN: A = −3.0 mm, L = +0.9 mm, V = −6.2 mm, relative to bregma) and reticular thalamic nucleus (RTN: A = −1.4 mm, L = +1.2 mm, V = −7.2 mm, relative to bregma) [65].

Perfusion experiments were started 18 h after recovery from isoflurane anaesthesia. The perfusion rate was set at 2 μL/min in all experiments, using modified Ringer’s solution (MRS) composed of the following (in mM): 145 NaCl, 2.7 KCl, 1.2 CaCl_2_, and 1.0 MgCl_2_, buffered with 2 mM phosphate buffer and 1.1 mM Tris buffer at pH 7.4. To activate hemichannel activity, perfusion medium was switched from MRS to Ca^2+^ free with 100 mM K^+^ containing MRS (FCHK-MRS): NaCl (49.1), KCl (100.0), and MgCl_2_ (1.0), buffered with 2 mM phosphate buffer and 1.1 mM Tris buffer at pH 7.4 for 20 min [60,66,67].

### 4.3. Experimental Designs of Microdialysis Study

Each rat was randomly assigned to the treatment groups of each experiment. The experiment was started after the coefficient of variation of the levels of transmitter reached less than 5% (stabilisation). After the confirming the stabilisation, perfusate was collected for 60 min (pretreatment period) followed by 180 min of sampling after AMPA-evoked stimulation.

**Figure 10:**
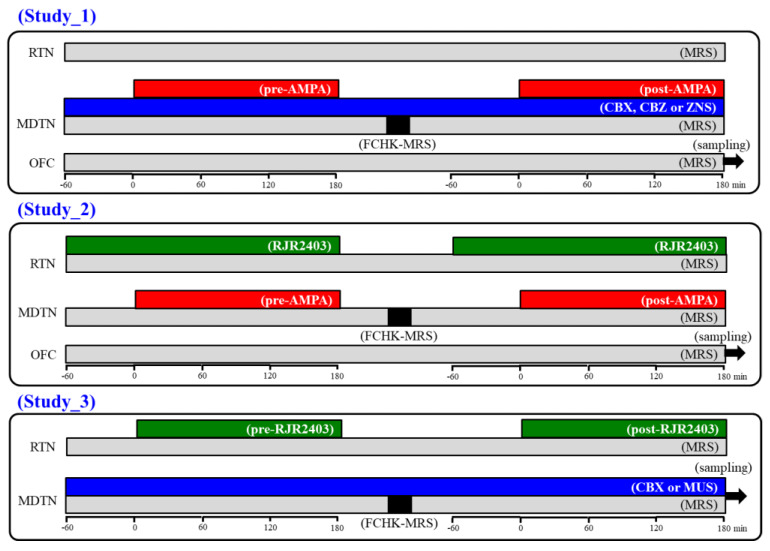
Schematic experimental designs of microdialysis. MRS: modified Ringer’s solution (gray columns), FCHK-MRS: Ca^2+^-free with 100 mM K^+^ containing MRS (closed columns), RJR2403 (100 μM: selective α4β2-nAChR agonist): (E)-*N*-Methyl-4-(3-pyridinyl)-3-buten-1-amine oxalate, AMPA (100 μM: AMPA/glutamate receptor agonist): amino-3-(3-hydroxy-5-methyl-isoxazol-4-yl) propanoic acid, CBX (100 μM: non-selective hemichannel inhibitor): carbenoxolone, CBZ (100 μM): carbamazepine, MDTN: mediodorsal thalamic nuclei, RTN: reticular thalamic nucleus, OFC: orbitofrontal cortex, and ZNS (500 μM): zonisamide.

It has been well known that, during the resting stage, the hemichannel is a low opening probability, but the extracellular cation condition, as well as increased K^+^ and decreased Ca^2+^ levels, activate hemichannel activity [40,41]. Using the primary cultured astrocytes study, 100 mM K^+^-evoked stimulation generated the activation of astroglial hemichannel, but 50 mM K^+^-evoked stimulation had no effect [29]. Using the microdialysis study, 100 mM K^+^-evoked stimulation also generated the prolonged activation of astroglial hemichannel (for several hours order) around the microdialysis probe [23]. According to previous demonstrations, in order to activate hemichannel activity, perfusion medium in the MDTN was changed to Ca^2+^-free with 100 mM K^+^ containing MRS for 20 min (FCHK-MRS activation) [23,29]. The schematic experimental designs are indicated in Figure 10.

#### 4.3.1. Study 1: Effects of CBX and Therapeutic-Relevant Concentrations of CBZ and ZNS on Glutamatergic Transmission in Thalamocortical Pathway

To explore the effects of hemichannel and anticonvulsants on glutamatergic transmission in the thalamocortical pathway (MDTN-M2C) of wild-type and S286L-TG, the perfusion medium in the MDTN was commenced with MRS with or without 100 μM CBX (non-selective hemichannel inhibitor), therapeutic-relevant concentration of CBZ (100 μM) and ZNS (500 μM). The perfusates in the MDTN and OFC were maintained with MRS alone during study 1. After the stabilisation of the l-glutamate level in the OFC, the perfusate in the MDTN was switched to MRS containing the same agent with 100 μM AMPA for 180 min (pre-AMPA stimulation). After the pre-AMPA stimulation, perfusion medium in the MDTN was switched to MRS. After the stabilisation of the l-glutamate level in the OFC, the perfusion medium in the MDTN was switched to FCHK-MRS for 20 min (hemichannel activation). After the stabilisation of the l-glutamate level in the OFC, perfusion medium in the MDTN was switched to MRS containing the same agent with 100 μM AMPA for 180 min again (post-AMPA stimulation) (Figure 10). The interval between pre- and post-AMPA stimulation was around 240 min.

#### 4.3.2. Study 2: Effects of RJR2403 on Glutamatergic Transmission in Thalamocortical Pathway

To explore the effects of α4β2-nAChR in the RTN on transmission in the thalamocortical pathway (RTN-MDTN-M2C) of wild-type and S286L-TG, the perfusion mediums in the RTN, MDTN, and OFC were commenced with MRS. After the stabilisation of the l-glutamate level in the OFC, the perfusate in the RTN was switched to MRS containing 100 μM RJR2403 (selective α4β2-nAChR agonist). After 60 min starting perfusion with RJR2403 into the RTN, perfusion medium in the MDTN was switched to MRS containing 100 μM AMPA for 180 min (pre-AMPA stimulation). After the pre-AMPA stimulation, perfusion mediums in the RTN and MDTN were switched to MRS. After the stabilization of the l-glutamate level in the OFC, the perfusion medium in the MDTN was switched to FCHK-MRS for 20 min (hemichannel activation). After the stabilisation of the l-glutamate level in the OFC, perfusion medium in the RTN was switched to MRS containing 100 μM RJR2403. After 60 min starting perfusion RJR2403 into the RTN, the perfusion medium into the MDTN was switched from MRS to MRS containing 100 μM AMPA for 180 min (post-AMPA stimulation). During the experiment, the perfusate in the OFC was maintained by MRS alone (Figure 10). The interval between pre- and post-AMPA stimulation was around 240 min.

#### 4.3.3. Study 3: Effects of α4β2-nAChR in the RTN on Intrathalamic Transmission

To explore the effects of α4β2-nAChR in the RTN on intrathalamic transmission (RTN-MDTN) of wild-type and S286L-TG, the perfusion mediums in the RTN and MDTN were commenced with MRS and MRS containing 100 μM CBX or 1 μM MUS, respectively. After the stabilisation of levels of l-glutamate and GABA in the MDTN, the perfusion medium in the RTN was switched to MRS containing 100 μM RJR2403 for 180 min (pre-RJR stimulation). After the pre-RJR stimulation, the perfusion medium in the RTN was switched to MRS. After the stabilization of the levels of l-glutamate and GABA the MDTN, the perfusion medium in the MDTN was switched to FCHK-MRS for 20 min (hemichannel activation). After the stabilisation of the levels of l-glutamate and GABA in the MDTN, the perfusion medium in the RTN was switched to MRS containing 100 μM RJR2403 for 180 min again (post-RJR stimulation) (Figure 10). The interval between pre- and post-RJR stimulation was around 240 min.

### 4.4. Ultra-High-Performance Liquid-Chromatography (UHPLC)

Extracellular levels of l-glutamate and GABA were determined by UHPLC equipped with xLC3185PU (Jasco, Tokyo, Japan) and fluorescence detector (xLC3120FP, Jasco) following dual derivatisation with isobutyryl-L-cysteine and o-phthalaldehyde [47,68]. Derivatised solutions were prepared by dissolving isobutyryl-L-cysteine (2 mg) and o-phthalaldehyde (2 mg) in 0.1 mL ethanol, followed by the addition of 0.9 mL sodium borate buffer (0.2 M, pH 9.0) [53,69,70]. Pre-column derivatisation was performed by drawing 5 μL dialysate sample, standard, or blank solutions and 5 μL of derivatisation solution in a reaction vial and incubating for 5 min before injecting to the UHPLC system. The derivatised samples (5 μL aliquots) were injected by an auto sampler (xLC3059AS, Jasco). The analytical column (YMC Triat C18, particle 1.8 μm, 50 × 2.1 mm, YMC, Kyoto, Japan) was maintained at 45 °C. A linear gradient elution programme was performed over a period of 10 min with mobile phases A (0.05 M citrate buffer, pH 5.0) and B (0.05 M citrate buffer containing 30% acetonitrile and 30% methanol, pH 3.5). The excitation/emission wavelengths of the fluorescence detector were set at 280/455 nm [53,69,70]. The flow rate was set at 500 μL/min.

### 4.5. Simple Western Analysis

Total proteins in cytosol and plasma membrane fractions of rat thalamus after subchronic administration of 25 mg/kg/day of ZNS and CBZ for seven days were extracted by Minute Plasma Membrane Protein Isolation Kit (Invent Biotechnologies, Plymouth, MN, USA) [29]. Simple Western analysis was performed by Wes instrument (ProteinSimple, Santa Clara, CA, USA) according to the ProteinSimple user manual [21,29]. Antibodies of glyceraldehyde-3-phosphate dehydrogenase (GAPDH) (NB300-322SS, Novus Biologicals, Littleton, CO, USA) and Cx43 (C6219, Sigma-Aldrich, St. Louis, MO, USA) were diluted in antibody diluent II (ProteinSimple) with 1:100 dilution. The digital images were analysed and quantified Compass for SW ver.4.1.0 (ProteinSimple).

### 4.6. Data Analysis

All experiments in this study were designed with equally sized animal groups (N = 6) without carrying out a formal power analysis, in keeping with previous studies [21,22,23,26,29,44,45,54]. All values are expressed as mean ± standard deviation (SD) and *p* < 0.05 (two-tailed) was considered statistically significant for all tests. Target agents’ concentrations in acutely local and subchronically systemic administrations were selected based on values in previous studies [21,22,26,29,44,45,54]. Where possible, we sought to randomise and blind the data. In particular, for the determination of extracellular transmitter levels, the sample order on the autosampler was determined by a random number table.

Extracellular transmitter levels were analysed by Mauchly’s sphericity test followed by multivariate analysis of variance (MANOVA) using BellCurve for Excel ver. 3.2 (Social Survey Research Information Co., Ltd., Tokyo, Japan). When the data did not violate the assumption of sphericity (*p* > 0.05), the F-value of MANOVA was analysed using sphericity assumed degrees of freedom. On the contrary, when the assumption of sphericity was violated (*p* < 0.05), the F-value was analysed using Chi-Muller’s corrected degrees of freedom. When the F-values for the genotype/drug/time factors of MANOVA were significant, the data were analysed by Tukey’s post hoc test. The transmitter level was expressed as the area under the curve between 20 and 180 min (AUC20–180) after perfusion of the target agent.

Expression of Cx43 of cytosol and plasma membrane fractions in thalamus was analysed by Student’s t-test or one-way analysis of variance (ANOVA) with Tukey’s post hoc test using BellCurve for Excel. All statistical analyses complied with the recommendations on experimental design and analysis in pharmacology [71].

### 4.7. Nomenclature of Targets and Ligands

Key protein targets and ligands in this article are hyperlinked to corresponding entries in http://www.guidetopharmacology.org, which is the common portal for data from the IUPHAR/BPS Guide to PHARMACOLOGY [72], and are permanently archived in the Concise Guide to PHARMACOLOGY 2017/18 [73].

## 5. Conclusions

This study provided the pathomechanism and pathophysiology of CBZ-resistant/ZNS-sensitive seizures and comorbid cognitive deficits of ADSHE with S284L-mutation using a valid ADSHE rat model, S286L-TG. Loss-of-function of S286L-mutant α4β2-nAChR in the RTN reduced GABAergic transmission in intrathalamic transmission (RTN-MDTN) and upregulated astroglial Cx43 expression in the thalamus and frontal cortex. The thalamic hemichannel consisting of upregulated astroglial Cx43 is activated (opening) during the resting stage. The combination of GABAergic disinhibition and activated hemichannel in the MDTN leads to hyperactivation of thalamocortical glutamatergic transmission, resulting in generation of epileptic hyperexcitability in the frontal cortex. Furthermore, hyperactivation of hemichannel composed of upregulated Cx43 in the MDTN loses the proper integration of signalling associated with cognition. ZNS chronically reduced thalamic Cx43 expression, and acutely supressed thalamocortical glutamatergic transmission via inhibition of thalamic hemichannel activity, whereas subchronic and acute administration of CBZ did not affect thalamic Cx43 expression and hemichannel activity. These results indicate the possible mechanisms of pathomechanisms and pathophysiology of ADSHE seizure and cognitive deficits of ADSHE with S286L-mutation.
